# Octa­kis(3-methyl­anilinium) hexa­chlorido­cadmate tetra­chloride

**DOI:** 10.1107/S1600536811049464

**Published:** 2011-11-25

**Authors:** Ming-Liang Liu

**Affiliations:** a College of Chemistryand Chemical Engineering, Southeast University, Nanjing 211189, People’s Republic of China

## Abstract

The asymmetric unit of the title compound, (C_7_H_10_N)_8_[CdCl_6_]Cl_4_, contains four 3-methyl­anilinium cations, two chloride anions and half an octa­hedral hexa­chloridocadmate(II) anion, which lies on an inversion centre. In the crystal, numerous N—H⋯Cl and bifurcated N—H⋯(Cl,Cl) hydrogen bonds link the components.

## Related literature

For background to ferroelectric metal-organic complexes, see: Ye *et al.* (2009 ▶); Zhang *et al.* (2009 ▶, 2010 ▶). For a related structure, see: Liu (2011 ▶).
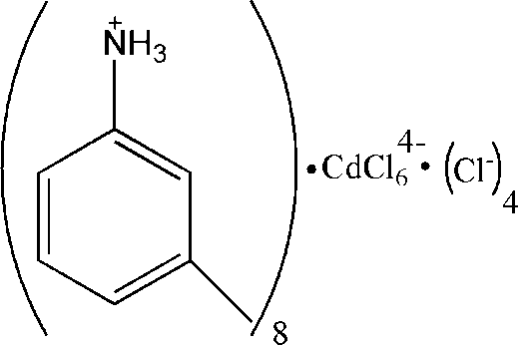


## Experimental

### 

#### Crystal data

(C_7_H_10_N)_8_[CdCl_6_]Cl_4_
*M* *_r_* = 1332.18
Triclinic, 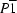

*a* = 8.8863 (18) Å
*b* = 14.116 (3) Å
*c* = 14.251 (3) Å
α = 87.92 (3)°
β = 71.88 (3)°
γ = 75.20 (3)°
*V* = 1640.8 (6) Å^3^
*Z* = 1
Mo *K*α radiation
μ = 0.78 mm^−1^
*T* = 293 K
0.20 × 0.20 × 0.20 mm

#### Data collection

Rigaku SCXmini CCD diffractometer
Absorption correction: multi-scan (*CrystalClear*; Rigaku, 2005 ▶) *T*_min_ = 0.860, *T*_max_ = 0.860
14045 measured reflections
5776 independent reflections
4134 reflections with *I* > 2σ(*I*)
*R*_int_ = 0.064

#### Refinement

*R*[*F* ^2^ > 2σ(*F* ^2^)] = 0.053
*wR*(*F* ^2^) = 0.144
*S* = 0.91
5776 reflections
348 parameters
18 restraints
H-atom parameters constrained
Δρ_max_ = 0.54 e Å^−3^
Δρ_min_ = −0.39 e Å^−3^

### 

Data collection: *CrystalClear* (Rigaku, 2005 ▶); cell refinement: *CrystalClear*; data reduction: *CrystalClear*; program(s) used to solve structure: *SHELXS97* (Sheldrick, 2008 ▶); program(s) used to refine structure: *SHELXL97* (Sheldrick, 2008 ▶); molecular graphics: *SHELXTL* (Sheldrick, 2008 ▶); software used to prepare material for publication: *SHELXTL*.

## Supplementary Material

Crystal structure: contains datablock(s) I, global. DOI: 10.1107/S1600536811049464/hb6519sup1.cif

Structure factors: contains datablock(s) I. DOI: 10.1107/S1600536811049464/hb6519Isup2.hkl

Additional supplementary materials:  crystallographic information; 3D view; checkCIF report

## Figures and Tables

**Table 1:** Selected bond lengths (Å)

Cd1—Cl1	2.5425 (12)
Cd1—Cl2	2.6743 (13)
Cd1—Cl3	2.6760 (15)

**Table 2:** Hydrogen-bond geometry (Å, °)

*D*—H⋯*A*	*D*—H	H⋯*A*	*D*⋯*A*	*D*—H⋯*A*
N1—H1*A*⋯Cl5^i^	0.89	2.38	3.264 (5)	176
N1—H1*B*⋯Cl4^ii^	0.89	2.47	3.309 (5)	157
N1—H1*C*⋯Cl2^iii^	0.89	2.54	3.329 (5)	148
N1—H1*C*⋯Cl3^iv^	0.89	2.88	3.427 (5)	121
N2—H2*A*⋯Cl4^v^	0.89	2.38	3.265 (5)	176
N2—H2*B*⋯Cl3^v^	0.89	2.51	3.300 (5)	149
N2—H2*B*⋯Cl2^vi^	0.89	2.93	3.495 (5)	123
N2—H2*C*⋯Cl5^vii^	0.89	2.33	3.186 (5)	162
N3—H3*A*⋯Cl4	0.89	2.37	3.254 (5)	172
N3—H3*B*⋯Cl3^vii^	0.89	2.61	3.344 (5)	140
N3—H3*B*⋯Cl2^vii^	0.89	2.75	3.367 (5)	127
N3—H3*C*⋯Cl5^vi^	0.89	2.44	3.289 (5)	159
N4—H4*A*⋯Cl4	0.89	2.40	3.270 (5)	167
N4—H4*B*⋯Cl5^vi^	0.89	2.38	3.267 (5)	177
N4—H4*C*⋯Cl2	0.89	2.55	3.305 (5)	143
N4—H4*C*⋯Cl3	0.89	2.91	3.526 (5)	128

Symmetry codes: (i) 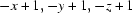; (ii) 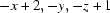; (iii) 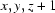; (iv) 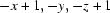; (v) 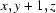; (vi) 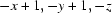; (vii) 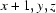.

**Fig. 1.**
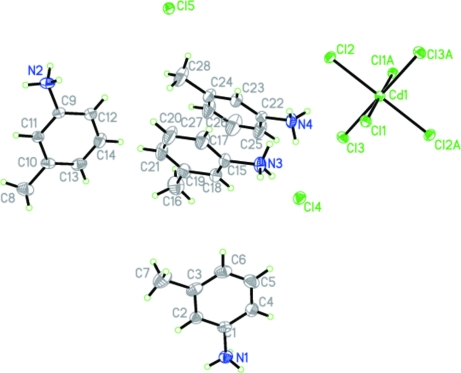
The molecular structure of the title compound, showing 30% probability displacement ellipsoids.

**Fig. 2.**
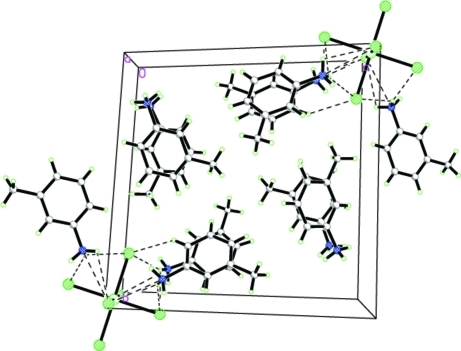
Crystal structure of the title compound with view along the a axis. Intermolecular interactions are shown as dashed lines.

## supplementary crystallographic information

### Comment

Recently much attention has been devoted to simple molecular-ionic compounds containing inorganic and organic ions due to the tunability of their special structural features and their potential ferroelectrics property. Ferroelectric materials that exhibit reversible electric polarization in response to an external electric field have found many applications such as nonvolatile memory storage, electronics and optics. The freezing of a certain functional group at low temperature forces significant orientational motions of the guest molecules and thus induces the formation of the ferroelectric phase. (Zhang *et al.* 2009; Ye *et al.* 2009; Zhang *et al.* 2010.). In our laboratory, the title compound, (I), has been synthesized and its crystal structure is herein reported.

The title compound, [(C_7_H_10_N)_8_CdCl_6_]Cl_4_, has an asymmertic unit that consists of four 3-methylanilinium cations, two chloride anions and one hexachloridocadmiumate anion (Fig 1), which lies in a symmetrical center. The non-hydrogen atoms of C_7_H_10_N cations are nearly coplanar, the cadmium atom is coordinated by six chloride ions, forming a distorted octahedron, the average Cd—Cl bond distances range from 2.5425 (12) Å to 2.6760 (15) Å, the Cl—Cd—Cl angles range from 88.87 (5)°to 180°.The existence of N—H···Cl hydrogen-bonding interactions makes great contribution to the stability of the crystal structure (Fig 2).

### Experimental

3.21 g (0.03 mol) of 3-methylbenzenamine was firstly dissolved in 30 ml ethanol, to which 1.1 g (0.03 mol) of hydrochloric acid was then added to afford the solution, then the 0.83 g (0.01 mol) cadmium chloride was dissolved in 20 ml e thanol which was added hydrochloric acid, at last, mixed the above solution without any precipitation under stirring at the ambient temperature. Single crystals suitable for X-ray structure analysis were obtained by the slow evaporation of the above solution after 4 days in air.

The dielectric constant of the compound as a function of temperature indicates that the permittivity is basically temperature-independent (*ε* = C/(T–T_0_)), suggesting that this compound is not ferroelectric or there may be no distinct phase transition occurring within the measured temperature within the measured temperature (below the melting point).

### Refinement

H atoms were placed in calculated positions (N—H = 0.89 Å; C—H = 0.93Å for C*sp^2^* atoms and C—H = 0.96Å and 0.97Å for C*sp^3^* atoms), assigned fixed *U*_iso_ values [*U*_iso_ = 1.2*U*eq(C*sp^2^*) and 1.5*U*eq(C*sp^3^*,*N*)] and allowed to ride.

### Crystal data

**Table:**

(C_7_H_10_N)_8_[CdCl_6_]Cl_4_	*V* = 1640.8 (6) Å^3^
*M**_r_* = 1332.18	*Z* = 1
Triclinic, *P*1	*F*(000) = 690
Hall symbol: -P 1	*D*_x_ = 1.348 Mg m^−^^3^
*a* = 8.8863 (18) Å	Mo *K*α radiation, λ = 0.71073 Å
*b* = 14.116 (3) Å	θ = 3.4–25°
*c* = 14.251 (3) Å	µ = 0.78 mm^−^^1^
α = 87.92 (3)°	*T* = 293 K
β = 71.88 (3)°	Block, colourless
γ = 75.20 (3)°	0.20 × 0.20 × 0.20 mm

### Data collection

**Table:**

Rigaku SCXmini CCD diffractometer	5776 independent reflections
Radiation source: fine-focus sealed tube	4134 reflections with *I* > 2σ(*I*)
graphite	*R*_int_ = 0.064
CCD_Profile_fitting scans	θ_max_ = 25.0°, θ_min_ = 3.0°
Absorption correction: multi-scan (*CrystalClear*; Rigaku, 2005)	*h* = −10→10
*T*_min_ = 0.860, *T*_max_ = 0.860	*k* = −16→16
14045 measured reflections	*l* = −16→16

### Refinement

**Table:**

Refinement on *F*^2^	Primary atom site location: structure-invariant direct methods
Least-squares matrix: full	Secondary atom site location: difference Fourier map
*R*[*F*^2^ > 2σ(*F*^2^)] = 0.053	Hydrogen site location: inferred from neighbouring sites
*wR*(*F*^2^) = 0.144	H-atom parameters constrained
*S* = 0.91	*w* = 1/[σ^2^(*F*_o_^2^) + (0.0846*P*)^2^ + 0.9416*P*] where *P* = (*F*_o_^2^ + 2*F*_c_^2^)/3
5776 reflections	(Δ/σ)_max_ = 0.074
348 parameters	Δρ_max_ = 0.54 e Å^−^^3^
18 restraints	Δρ_min_ = −0.39 e Å^−^^3^

### Special details

**Table:**

Geometry. All esds (except the esd in the dihedral angle between two l.s. planes) are estimated using the full covariance matrix. The cell esds are taken into account individually in the estimation of esds in distances, angles and torsion angles; correlations between esds in cell parameters are only used when they are defined by crystal symmetry. An approximate (isotropic) treatment of cell esds is used for estimating esds involving l.s. planes.
Refinement. Refinement of F^2^ against ALL reflections. The weighted R-factor wR and goodness of fit S are based on F^2^, conventional R-factors R are based on F, with F set to zero for negative F^2^. The threshold expression of F^2^ > 2sigma(F^2^) is used only for calculating R-factors(gt) etc. and is not relevant to the choice of reflections for refinement. R-factors based on F^2^ are statistically about twice as large as those based on F, and R- factors based on ALL data will be even larger.

### Fractional atomic coordinates and isotropic or equivalent isotropic displacement parameters (Å^2^)

**Table:**

	*x*	*y*	*z*	*U*_iso_*/*U*_eq_	
N1	0.8397 (6)	0.1086 (3)	0.7966 (3)	0.0507 (11)	
H1A	0.8672	0.1473	0.8329	0.076*	
H1B	0.9020	0.0477	0.7935	0.076*	
H1C	0.7349	0.1089	0.8241	0.076*	
C1	0.8646 (6)	0.1450 (4)	0.6958 (4)	0.0443 (13)	
C2	0.9124 (6)	0.2302 (4)	0.6733 (4)	0.0483 (13)	
H2	0.9252	0.2660	0.7225	0.058*	
C3	0.9416 (7)	0.2637 (4)	0.5790 (4)	0.0578 (16)	
C4	0.8444 (7)	0.0901 (4)	0.6257 (4)	0.0592 (15)	
H4	0.8137	0.0318	0.6416	0.071*	
C5	0.8702 (8)	0.1226 (5)	0.5312 (5)	0.0686 (18)	
H5	0.8537	0.0874	0.4830	0.082*	
C6	0.9207 (8)	0.2074 (5)	0.5084 (4)	0.0665 (18)	
H6	0.9414	0.2276	0.4439	0.080*	
C7	0.9997 (10)	0.3560 (5)	0.5545 (5)	0.087 (2)	
H7A	1.0879	0.3451	0.4933	0.130*	
H7B	1.0374	0.3729	0.6063	0.130*	
H7C	0.9109	0.4086	0.5483	0.130*	
C8	0.6267 (8)	0.8805 (5)	0.5669 (4)	0.0725 (19)	
H8A	0.5254	0.8858	0.6189	0.109*	
H8B	0.6492	0.9438	0.5575	0.109*	
H8C	0.7139	0.8355	0.5842	0.109*	
N2	0.7149 (5)	0.9027 (3)	0.2064 (3)	0.0496 (11)	
H2A	0.7556	0.9510	0.2179	0.074*	
H2B	0.6314	0.9273	0.1834	0.074*	
H2C	0.7925	0.8593	0.1620	0.074*	
C9	0.6575 (6)	0.8539 (4)	0.2994 (4)	0.0389 (12)	
C10	0.6136 (7)	0.8436 (4)	0.4724 (4)	0.0495 (14)	
C11	0.6709 (6)	0.8869 (4)	0.3835 (4)	0.0421 (12)	
H11	0.7186	0.9387	0.3820	0.050*	
C12	0.5901 (7)	0.7776 (4)	0.2968 (4)	0.0546 (15)	
H12	0.5832	0.7556	0.2380	0.065*	
C13	0.5448 (7)	0.7675 (5)	0.4711 (4)	0.0608 (16)	
H13	0.5058	0.7375	0.5297	0.073*	
C14	0.5323 (8)	0.7343 (5)	0.3850 (5)	0.0681 (18)	
H14	0.4848	0.6826	0.3859	0.082*	
C16	0.7077 (9)	0.4512 (5)	0.3820 (5)	0.094 (2)	
H16A	0.7335	0.4948	0.4213	0.140*	
H16B	0.6992	0.3919	0.4159	0.140*	
H16C	0.6054	0.4822	0.3711	0.140*	
C15	1.0231 (6)	0.3114 (4)	0.1562 (4)	0.0459 (13)	
N3	1.0857 (5)	0.2101 (3)	0.1154 (3)	0.0500 (11)	
H3A	1.0286	0.1724	0.1545	0.075*	
H3B	1.1908	0.1885	0.1115	0.075*	
H3C	1.0758	0.2079	0.0553	0.075*	
C17	1.0827 (8)	0.3832 (5)	0.1010 (5)	0.0707 (18)	
H17	1.1632	0.3677	0.0399	0.085*	
C18	0.9049 (7)	0.3321 (4)	0.2458 (4)	0.0504 (14)	
H18	0.8673	0.2816	0.2812	0.061*	
C19	0.8402 (7)	0.4276 (4)	0.2847 (5)	0.0587 (16)	
C20	1.0205 (10)	0.4790 (5)	0.1385 (6)	0.086 (2)	
H20	1.0587	0.5290	0.1026	0.103*	
C21	0.9007 (9)	0.5002 (5)	0.2298 (6)	0.075 (2)	
H21	0.8601	0.5649	0.2548	0.090*	
N4	0.6578 (6)	0.2081 (3)	0.1049 (4)	0.0551 (12)	
H4A	0.6956	0.1702	0.1481	0.083*	
H4B	0.7416	0.2172	0.0548	0.083*	
H4C	0.5971	0.1793	0.0821	0.083*	
C22	0.5575 (7)	0.3031 (4)	0.1537 (4)	0.0458 (13)	
C23	0.5977 (7)	0.3871 (4)	0.1165 (4)	0.0511 (14)	
H23	0.6900	0.3836	0.0620	0.061*	
C24	0.5009 (8)	0.4776 (4)	0.1602 (5)	0.0579 (15)	
C25	0.4232 (10)	0.3057 (5)	0.2339 (5)	0.088 (2)	
H25	0.3969	0.2481	0.2584	0.105*	
C26	0.3282 (11)	0.3950 (6)	0.2776 (6)	0.112 (2)	
H26	0.2360	0.3982	0.3321	0.134*	
C27	0.3685 (10)	0.4804 (5)	0.2410 (5)	0.088 (2)	
H27	0.3042	0.5406	0.2720	0.105*	
C28	0.5396 (11)	0.5721 (5)	0.1201 (6)	0.098 (3)	
H28A	0.4503	0.6266	0.1526	0.147*	
H28B	0.5553	0.5720	0.0503	0.147*	
H28C	0.6377	0.5777	0.1319	0.147*	
Cd1	0.5000	0.0000	0.0000	0.03328 (17)	
Cl1	0.78766 (14)	−0.00243 (8)	−0.00119 (8)	0.0353 (3)	
Cl2	0.45880 (15)	0.18683 (8)	−0.05106 (9)	0.0418 (3)	
Cl3	0.38016 (15)	0.06130 (9)	0.19060 (9)	0.0436 (3)	
Cl4	0.84395 (16)	0.08670 (9)	0.25439 (10)	0.0476 (3)	
Cl5	0.04194 (17)	0.75072 (10)	0.07936 (10)	0.0501 (3)	

### Atomic displacement parameters (Å^2^)

**Table:**

	*U*^11^	*U*^22^	*U*^33^	*U*^12^	*U*^13^	*U*^23^
N1	0.053 (3)	0.055 (3)	0.044 (3)	−0.020 (2)	−0.012 (2)	0.013 (2)
C1	0.036 (3)	0.055 (3)	0.035 (3)	−0.005 (2)	−0.006 (2)	0.008 (2)
C2	0.053 (3)	0.048 (3)	0.041 (3)	−0.011 (3)	−0.012 (3)	0.006 (3)
C3	0.048 (3)	0.058 (4)	0.050 (4)	−0.003 (3)	−0.002 (3)	0.017 (3)
C4	0.070 (4)	0.050 (4)	0.057 (4)	−0.013 (3)	−0.020 (3)	0.003 (3)
C5	0.078 (5)	0.077 (5)	0.046 (4)	−0.007 (4)	−0.023 (3)	−0.007 (3)
C6	0.069 (4)	0.073 (5)	0.039 (3)	0.005 (3)	−0.010 (3)	0.007 (3)
C7	0.093 (5)	0.079 (5)	0.078 (5)	−0.032 (4)	−0.007 (4)	0.034 (4)
C8	0.081 (5)	0.086 (5)	0.043 (4)	−0.011 (4)	−0.017 (3)	0.000 (3)
N2	0.051 (3)	0.062 (3)	0.035 (2)	−0.015 (2)	−0.014 (2)	0.012 (2)
C9	0.037 (3)	0.040 (3)	0.034 (3)	−0.005 (2)	−0.008 (2)	0.008 (2)
C10	0.049 (3)	0.059 (4)	0.035 (3)	−0.006 (3)	−0.013 (3)	0.005 (3)
C11	0.046 (3)	0.044 (3)	0.035 (3)	−0.011 (2)	−0.012 (2)	0.004 (2)
C12	0.069 (4)	0.044 (3)	0.056 (4)	−0.011 (3)	−0.031 (3)	0.005 (3)
C13	0.063 (4)	0.070 (4)	0.048 (4)	−0.024 (3)	−0.013 (3)	0.023 (3)
C14	0.082 (5)	0.050 (4)	0.082 (5)	−0.029 (3)	−0.031 (4)	0.015 (3)
C16	0.101 (6)	0.077 (5)	0.075 (5)	0.010 (4)	−0.010 (4)	−0.032 (4)
C15	0.050 (3)	0.033 (3)	0.051 (3)	−0.007 (2)	−0.014 (3)	−0.003 (2)
N3	0.049 (3)	0.038 (3)	0.057 (3)	−0.006 (2)	−0.012 (2)	−0.010 (2)
C17	0.079 (5)	0.052 (4)	0.072 (4)	−0.023 (3)	−0.006 (4)	0.003 (3)
C18	0.056 (3)	0.040 (3)	0.053 (3)	−0.007 (3)	−0.018 (3)	−0.002 (3)
C19	0.063 (4)	0.046 (4)	0.060 (4)	0.004 (3)	−0.022 (3)	−0.013 (3)
C20	0.113 (6)	0.045 (4)	0.101 (6)	−0.035 (4)	−0.026 (5)	0.021 (4)
C21	0.092 (5)	0.035 (4)	0.092 (5)	−0.004 (3)	−0.027 (4)	−0.015 (3)
N4	0.065 (3)	0.034 (3)	0.069 (3)	−0.009 (2)	−0.027 (3)	−0.005 (2)
C22	0.055 (3)	0.035 (3)	0.051 (3)	−0.006 (2)	−0.026 (3)	0.003 (2)
C23	0.052 (3)	0.041 (3)	0.052 (3)	−0.011 (3)	−0.005 (3)	0.002 (3)
C24	0.067 (4)	0.040 (3)	0.065 (4)	−0.012 (3)	−0.019 (3)	0.009 (3)
C25	0.115 (5)	0.048 (4)	0.069 (4)	−0.027 (4)	0.021 (4)	0.000 (3)
C26	0.117 (5)	0.075 (4)	0.095 (4)	−0.020 (4)	0.030 (4)	−0.006 (4)
C27	0.097 (5)	0.046 (4)	0.087 (5)	−0.006 (4)	0.010 (4)	−0.019 (3)
C28	0.119 (7)	0.051 (4)	0.121 (7)	−0.036 (4)	−0.023 (6)	0.019 (4)
Cd1	0.0356 (3)	0.0329 (3)	0.0336 (3)	−0.0111 (2)	−0.0123 (2)	0.0032 (2)
Cl1	0.0363 (6)	0.0349 (6)	0.0361 (6)	−0.0119 (5)	−0.0111 (5)	0.0029 (5)
Cl2	0.0473 (7)	0.0325 (7)	0.0476 (7)	−0.0118 (5)	−0.0174 (6)	0.0086 (5)
Cl3	0.0475 (7)	0.0479 (8)	0.0341 (7)	−0.0119 (6)	−0.0109 (6)	−0.0017 (5)
Cl4	0.0552 (8)	0.0414 (7)	0.0449 (7)	−0.0151 (6)	−0.0123 (6)	0.0069 (6)
Cl5	0.0612 (9)	0.0426 (8)	0.0430 (7)	−0.0103 (6)	−0.0136 (6)	−0.0006 (6)

### Geometric parameters (Å, °)

**Table:**

N1—C1	1.478 (6)	C16—H16C	0.9600
N1—H1A	0.8900	C15—C18	1.362 (7)
N1—H1B	0.8900	C15—C17	1.372 (8)
N1—H1C	0.8900	C15—N3	1.464 (6)
C1—C4	1.367 (8)	N3—H3A	0.8900
C1—C2	1.371 (7)	N3—H3B	0.8900
C2—C3	1.377 (7)	N3—H3C	0.8900
C2—H2	0.9300	C17—C20	1.380 (9)
C3—C6	1.387 (9)	C17—H17	0.9300
C3—C7	1.512 (9)	C18—C19	1.383 (7)
C4—C5	1.375 (8)	C18—H18	0.9300
C4—H4	0.9300	C19—C21	1.384 (9)
C5—C6	1.377 (9)	C20—C21	1.385 (10)
C5—H5	0.9300	C20—H20	0.9300
C6—H6	0.9300	C21—H21	0.9300
C7—H7A	0.9600	N4—C22	1.465 (7)
C7—H7B	0.9600	N4—H4A	0.8900
C7—H7C	0.9600	N4—H4B	0.8900
C8—C10	1.509 (8)	N4—H4C	0.8900
C8—H8A	0.9600	C22—C25	1.365 (8)
C8—H8B	0.9600	C22—C23	1.366 (7)
C8—H8C	0.9600	C23—C24	1.386 (8)
N2—C9	1.478 (6)	C23—H23	0.9300
N2—H2A	0.8900	C24—C27	1.359 (9)
N2—H2B	0.8900	C24—C28	1.510 (8)
N2—H2C	0.8900	C25—C26	1.369 (10)
C9—C11	1.351 (7)	C25—H25	0.9300
C9—C12	1.366 (7)	C26—C27	1.383 (10)
C10—C13	1.368 (8)	C26—H26	0.9300
C10—C11	1.393 (7)	C27—H27	0.9300
C11—H11	0.9300	C28—H28A	0.9600
C12—C14	1.385 (8)	C28—H28B	0.9600
C12—H12	0.9300	C28—H28C	0.9600
C13—C14	1.376 (9)	Cd1—Cl1^i^	2.5425 (12)
C13—H13	0.9300	Cd1—Cl1	2.5425 (12)
C14—H14	0.9300	Cd1—Cl2	2.6743 (13)
C16—C19	1.496 (9)	Cd1—Cl2^i^	2.6743 (13)
C16—H16A	0.9600	Cd1—Cl3	2.6760 (15)
C16—H16B	0.9600	Cd1—Cl3^i^	2.6760 (15)
			
C1—N1—H1A	109.5	C17—C15—N3	118.5 (5)
C1—N1—H1B	109.5	C15—N3—H3A	109.5
H1A—N1—H1B	109.5	C15—N3—H3B	109.5
C1—N1—H1C	109.5	H3A—N3—H3B	109.5
H1A—N1—H1C	109.5	C15—N3—H3C	109.5
H1B—N1—H1C	109.5	H3A—N3—H3C	109.5
C4—C1—C2	121.4 (5)	H3B—N3—H3C	109.5
C4—C1—N1	118.5 (5)	C15—C17—C20	118.4 (6)
C2—C1—N1	120.1 (5)	C15—C17—H17	120.8
C1—C2—C3	121.0 (5)	C20—C17—H17	120.8
C1—C2—H2	119.5	C15—C18—C19	120.6 (6)
C3—C2—H2	119.5	C15—C18—H18	119.7
C2—C3—C6	117.1 (6)	C19—C18—H18	119.7
C2—C3—C7	120.4 (6)	C18—C19—C21	117.7 (6)
C6—C3—C7	122.5 (6)	C18—C19—C16	121.0 (6)
C1—C4—C5	118.8 (6)	C21—C19—C16	121.3 (6)
C1—C4—H4	120.6	C17—C20—C21	119.7 (6)
C5—C4—H4	120.6	C17—C20—H20	120.1
C4—C5—C6	119.7 (6)	C21—C20—H20	120.1
C4—C5—H5	120.2	C19—C21—C20	121.6 (6)
C6—C5—H5	120.2	C19—C21—H21	119.2
C5—C6—C3	122.0 (6)	C20—C21—H21	119.2
C5—C6—H6	119.0	C22—N4—H4A	109.5
C3—C6—H6	119.0	C22—N4—H4B	109.5
C3—C7—H7A	109.5	H4A—N4—H4B	109.5
C3—C7—H7B	109.5	C22—N4—H4C	109.5
H7A—C7—H7B	109.5	H4A—N4—H4C	109.5
C3—C7—H7C	109.5	H4B—N4—H4C	109.5
H7A—C7—H7C	109.5	C25—C22—C23	121.5 (5)
H7B—C7—H7C	109.5	C25—C22—N4	119.1 (5)
C10—C8—H8A	109.5	C23—C22—N4	119.4 (5)
C10—C8—H8B	109.5	C22—C23—C24	120.0 (5)
H8A—C8—H8B	109.5	C22—C23—H23	120.0
C10—C8—H8C	109.5	C24—C23—H23	120.0
H8A—C8—H8C	109.5	C27—C24—C23	118.8 (5)
H8B—C8—H8C	109.5	C27—C24—C28	119.7 (6)
C9—N2—H2A	109.5	C23—C24—C28	121.5 (6)
C9—N2—H2B	109.5	C22—C25—C26	118.6 (6)
H2A—N2—H2B	109.5	C22—C25—H25	120.7
C9—N2—H2C	109.5	C26—C25—H25	120.7
H2A—N2—H2C	109.5	C25—C26—C27	120.3 (7)
H2B—N2—H2C	109.5	C25—C26—H26	119.8
C11—C9—C12	122.6 (5)	C27—C26—H26	119.8
C11—C9—N2	119.2 (4)	C24—C27—C26	120.8 (6)
C12—C9—N2	118.2 (5)	C24—C27—H27	119.6
C13—C10—C11	118.0 (5)	C26—C27—H27	119.6
C13—C10—C8	121.5 (5)	C24—C28—H28A	109.5
C11—C10—C8	120.5 (5)	C24—C28—H28B	109.5
C9—C11—C10	120.1 (5)	H28A—C28—H28B	109.5
C9—C11—H11	119.9	C24—C28—H28C	109.5
C10—C11—H11	119.9	H28A—C28—H28C	109.5
C9—C12—C14	117.6 (5)	H28B—C28—H28C	109.5
C9—C12—H12	121.2	Cl1^i^—Cd1—Cl1	180.0
C14—C12—H12	121.2	Cl1^i^—Cd1—Cl2	91.13 (5)
C10—C13—C14	121.3 (5)	Cl1—Cd1—Cl2	88.87 (5)
C10—C13—H13	119.3	Cl1^i^—Cd1—Cl2^i^	88.87 (5)
C14—C13—H13	119.3	Cl1—Cd1—Cl2^i^	91.13 (5)
C13—C14—C12	120.3 (6)	Cl2—Cd1—Cl2^i^	180.0
C13—C14—H14	119.8	Cl1^i^—Cd1—Cl3	89.47 (5)
C12—C14—H14	119.8	Cl1—Cd1—Cl3	90.53 (5)
C19—C16—H16A	109.5	Cl2—Cd1—Cl3	89.47 (5)
C19—C16—H16B	109.5	Cl2^i^—Cd1—Cl3	90.53 (5)
H16A—C16—H16B	109.5	Cl1^i^—Cd1—Cl3^i^	90.53 (5)
C19—C16—H16C	109.5	Cl1—Cd1—Cl3^i^	89.47 (5)
H16A—C16—H16C	109.5	Cl2—Cd1—Cl3^i^	90.53 (5)
H16B—C16—H16C	109.5	Cl2^i^—Cd1—Cl3^i^	89.47 (5)
C18—C15—C17	122.0 (5)	Cl3—Cd1—Cl3^i^	180.0
C18—C15—N3	119.5 (5)		
			
C4—C1—C2—C3	0.0 (8)	C18—C15—C17—C20	0.2 (10)
N1—C1—C2—C3	177.8 (5)	N3—C15—C17—C20	178.3 (6)
C1—C2—C3—C6	0.0 (8)	C17—C15—C18—C19	0.0 (9)
C1—C2—C3—C7	−177.9 (6)	N3—C15—C18—C19	−178.1 (5)
C2—C1—C4—C5	−1.1 (9)	C15—C18—C19—C21	−0.5 (9)
N1—C1—C4—C5	−178.8 (5)	C15—C18—C19—C16	178.6 (6)
C1—C4—C5—C6	2.1 (9)	C15—C17—C20—C21	0.0 (11)
C4—C5—C6—C3	−2.1 (10)	C18—C19—C21—C20	0.7 (10)
C2—C3—C6—C5	1.1 (9)	C16—C19—C21—C20	−178.3 (7)
C7—C3—C6—C5	178.9 (6)	C17—C20—C21—C19	−0.5 (12)
C12—C9—C11—C10	−0.6 (8)	C25—C22—C23—C24	−0.3 (9)
N2—C9—C11—C10	178.3 (5)	N4—C22—C23—C24	178.0 (5)
C13—C10—C11—C9	0.2 (8)	C22—C23—C24—C27	1.2 (9)
C8—C10—C11—C9	−179.4 (5)	C22—C23—C24—C28	−178.8 (6)
C11—C9—C12—C14	0.8 (8)	C23—C22—C25—C26	−0.2 (12)
N2—C9—C12—C14	−178.2 (5)	N4—C22—C25—C26	−178.5 (7)
C11—C10—C13—C14	0.0 (9)	C22—C25—C26—C27	−0.3 (14)
C8—C10—C13—C14	179.7 (6)	C23—C24—C27—C26	−1.7 (12)
C10—C13—C14—C12	0.2 (10)	C28—C24—C27—C26	178.4 (8)
C9—C12—C14—C13	−0.6 (9)	C25—C26—C27—C24	1.2 (15)

Symmetry codes: (i) −*x*+1, −*y*, −*z*.

### Hydrogen-bond geometry (Å, °)

**Table:**

*D*—H···*A*	*D*—H	H···*A*	*D*···*A*	*D*—H···*A*
N1—H1A···Cl5^ii^	0.89	2.38	3.264 (5)	176
N1—H1B···Cl4^iii^	0.89	2.47	3.309 (5)	157
N1—H1C···Cl2^iv^	0.89	2.54	3.329 (5)	148
N1—H1C···Cl3^v^	0.89	2.88	3.427 (5)	121
N2—H2A···Cl4^vi^	0.89	2.38	3.265 (5)	176
N2—H2B···Cl3^vi^	0.89	2.51	3.300 (5)	149
N2—H2B···Cl2^vii^	0.89	2.93	3.495 (5)	123
N2—H2C···Cl5^viii^	0.89	2.33	3.186 (5)	162
N3—H3A···Cl4	0.89	2.37	3.254 (5)	172
N3—H3B···Cl3^viii^	0.89	2.61	3.344 (5)	140
N3—H3B···Cl2^viii^	0.89	2.75	3.367 (5)	127
N3—H3C···Cl5^vii^	0.89	2.44	3.289 (5)	159
N4—H4A···Cl4	0.89	2.40	3.270 (5)	167
N4—H4B···Cl5^vii^	0.89	2.38	3.267 (5)	177
N4—H4C···Cl2	0.89	2.55	3.305 (5)	143
N4—H4C···Cl3	0.89	2.91	3.526 (5)	128

Symmetry codes: (ii) −*x*+1, −*y*+1, −*z*+1; (iii) −*x*+2, −*y*, −*z*+1; (iv) *x*, *y*, *z*+1; (v) −*x*+1, −*y*, −*z*+1; (vi) *x*, *y*+1, *z*; (vii) −*x*+1, −*y*+1, −*z*; (viii) *x*+1, *y*, *z*.
